# Intraoperative Protective Mechanical Ventilation in Dogs: A Randomized Clinical Trial

**DOI:** 10.3389/fvets.2022.842613

**Published:** 2022-03-15

**Authors:** Renata R. Rodrigues, Aline M. Ambrósio, Aline M. Engbruch, Lucas A. Gonçalves, Paula A. Villela, Ana F. Sanchez, Denise T. Fantoni

**Affiliations:** Department of Surgery, Faculty of Veterinary Medicine and Animal Science, University of São Paulo, São Paulo, Brazil

**Keywords:** mechanical ventilation, low tidal volume, alveolar recruitment maneuver, positive end-expiratory pressure, gas exchange, ventilatory mechanics, hemodynamics, dogs

## Abstract

**Objective:**

To evaluate gas exchange, respiratory mechanics, and hemodynamic impact of mechanical ventilation with low tidal volume (V_T_) in dogs with the use of positive end-expiratory pressure (PEEP) or preceded by alveolar recruitment maneuver (ARM).

**Study Design:**

Prospective randomized clinical trial.

**Animals:**

Twenty-one healthy client-owned mesocephalic healthy dogs, 1–7 years old, weighing 10–20 kg, and body condition scores 4–6/9 admitted for periodontal treatment.

**Methods:**

Isoflurane-anesthetized dogs in dorsal recumbency were ventilated until 1 h with a volume-controlled ventilation mode using 8 mL kg^−1^ of V_T_. The dogs were distributed in 2 groups: in the ARM group, PEEP starts in 0 cmH_2_O, increasing gradually 5 cmH_2_O every 3 min, until reach 15 cmH_2_O and decreasing in the same steps until 5 cmH_2_O, maintaining this value until the end; and PEEP group, in which the pressure 5 cmH_2_O was instituted from the beginning of anesthesia and maintained the same level up to the end of the anesthesia. Cardiopulmonary, metabolic, oxygenation parameters, and respiratory mechanics were recorded after the anesthesia induction (baseline—BL), 15, 45, and 75 min after BL and during the recovery.

**Results:**

The ARM increased the static compliance (C_st_) (15 min after baseline) when compared with baseline moment (24.9 ± 5.8 mL cmH_2_0^−1^ vs. 20.7 ± 5.4 mL cmH_2_0^−1^–*p* = 0.0364), oxygenation index (PaO_2_/FIO_2_) (505.6 ± 59.2 mmHg vs. 461.2 ± 41.0 mmHg—*p* = 0.0453) and reduced the shunt fraction (3.4 ± 2.4% vs. 5.5 ± 1.6%—*p* = 0.062). In the PEEP group, no statistical differences were observed concerning the variables evaluated. At the beginning of the evaluation, the driving pressure (DP) before ARM was significantly greater than all other evaluation time points (6.9 ± 1.8 cmH_2_0).

**Conclusions and Clinical Relevance:**

The use of 8 mL kg^−1^ of V_T_ and 5 cmH_2_0 PEEP without ARM maintain adequate oxygenation and mechanical ventilation in dental surgeries for up to 1 h. The use of ARM slightly improved compliance and oxygenation during the maneuver.

## Introduction

Perioperative lung-protective ventilation can be defined as the use of low tidal volume (V_T_) and plateau pressure (*P*_plat_), use of positive end-expiratory pressure (PEEP), and alveolar recruitment maneuvers (ARM) in human beings (1, 2). These intraoperative strategies are related to a reduced risk of post-operative pulmonary complications in healthy patients submitted to general anesthesia with mechanical ventilation. The main post-operative complications reported are respiratory failure, pneumonia, hypoxemia, atelectasis, bronchospasm, pleural effusion, pneumothorax, and ventilatory depression (2–5).

In humans healthy lungs, protective ventilation is achieved with low V_T_ (6–8 mL kg^−1^), maintenance of PEEP, inspired oxygen fraction (FIO_2_) to keep SpO_2_ at least 92%, respiratory rate (*f*_R_) to maintain end-tidal carbon dioxide (PE'CO_2_) between 35 and 50 mmHg (4.7–6.7 kPa) and ARM just when necessary (6–10).

However, according to the literature, in healthy patients, pulmonary complications are not prevented just by using lower V_T_. PEEP administration is recommended to avoid the cyclic opening and closing of the alveoli which, causes inflammation and lesion in the parenchyma, predisposing the alveoli collapse (11). Furthermore, alveolar recruitment aims to reopen closed areas of the lung, with the posterior application of PEEP capable of keeping it open, avoiding recollapse (12–14). To hold the alveoli open, besides using strategies as PEEP and ARM, the use of low FIO_2_ is necessary. Lung also suffers when submitted to high oxygen concentrations for long periods, resulting in tissue inflammation and pulmonary edema (15).

In healthy dogs, the last years literature mentions a V_T_ from 7 to 15 mL kg^−1^, with or without PEEP until 5 cmH_2_O (16–20). Some authors use recruitment maneuvers whether by PEEP titration (18), by increased inspiratory pressure (21), or both (20).

This study aimed to evaluate gas exchange, mechanical variables, and the hemodynamic changes in healthy dogs ventilated with low V_T_ and PEEP preceded by ARM or not. We hypothesize that a low volume of 8 mL kg^−1^ associated with PEEP 5 cmH_2_O and ARM would be able to ventilate dogs without promoting post-operative pulmonary complications properly.

## Materials and Methods

The Institutional Animal Use Ethics Committee approved the experimental protocol under Protocol No. 3581110716. Written owner consent was obtained before the entry of any dog into the study.

### Animals

In this study, a total of 21 clients' dogs of any breeds and gender were included. The minimum sample size for paired data was calculated using power analysis.^1^ Eight dogs per group would be required to detect a 95% chance (with 5% risk) of an increase in C_st_.kg^−1^ from 1.3 cmH_2_O.kg^−1^ in the treatment group and 1.8 cmH_2_O.kg^−1^ for the control group.

The inclusion criteria were 1–7 years old, body weight of 10–20 kg, body condition score from 4 to 6/9 (22), mesocephalic, with periodontal disease stage 1 and American Society of Anesthesiologists (ASA) risk classification of I or II and no known history of respiratory or cardiovascular diseases. All patients underwent periodontal treatment surgery without dental extraction of at least 1 h and in dorsal recumbency. Dogs were considered healthy based on anamnesis, physical examination, laboratory tests (blood count, serum biochemical, and arterial blood gas analysis), and electrocardiogram.

### Anesthesia and Monitoring

Dogs fasted for 8 h and water was withheld for 4 h. Premedication was accomplished with intramuscular injection (IM) of 0.03 mg kg^−1^ of acepromazine (Acepromazina; Syntec, Brazil) and meperidine 3 mg kg^−1^ (Dolosan; Cristália LTDA, Brazil). After 20 min, a 20 Gauge catheter (Angiocath; BD, Brazil) was placed in the cephalic vein, and anesthesia was induced with propofol (3–5 mg kg^−1^ Propovan; Cristália LTDA, Brazil) intravenously (IV) to achieve relaxation of the mandibular muscles and loss of the laryngeal reflex. After orotracheal intubation, the animals were placed in dorsal recumbency on a heated mat, and the endotracheal tube was connected to a rebreathing circuit with a microprocessor-controlled anesthesia ventilator (Fabius Plus; Drager, Germany). Anesthesia was maintained with end-tidal isoflurane (Isoforine; Cristália LTDA, Brazil) of (FE'Iso) 1.2–1.4% and FIO_2_ of 0.5 and monitored by a side stream non-dispersive infrared gas analyzer (POET IQ2-8500Q; Criticare Systems Inc., WI, USA) that was calibrated before each experiment.

Fluid therapy with lactated Ringer's solution (LR; Fresenius Kabi, Brazil) was administered at a constant rate infusion (CRI) of 5 mL kg^−1^ h^−1^. Neuromuscular blockade (NB) was instituted by IV rocuronium 0.6 mg kg^−1^ (Esmeron; Organon LTDA, Brazil), and a neuromuscular monitor in the train-of-four mode (TOF-Guard Biometer; Organon Teknika, Brazil) was used to ensure complete muscle paralysis.

The dorsal pedal artery was catheterized with a 22 Gauge catheter for continuous monitoring of mean arterial pressure (MAP) and arterial blood sampling for measurement of pH, arterial partial pressure of carbon dioxide (PaCO_2_) and of oxygen (PaO_2_), hemoglobin oxygen saturation (SaO_2_), base excess (BE) and bicarbonate (${\text{HCO}}_{3}^{-}$). Arterial blood samples were collected in a blood gas syringe (BD A-Line^®^ Beckton and Dickinson) and immediately analyzed (COBAS B121; Roché Diagnóstica, Brazil) and corrected for rectal temperature. The pressure transducer was placed at the level of the scapulohumeral joint and connected to a multiparametric data collection system (DX 2020; Dixtal, Brazil) for continuous monitoring of arterial pressure (mean—MAP, systolic—SAP, and diastolic—DAP) and waveforms. The same monitor provided the heart rate (HR) and rhythm by continuous electrocardiography. The cardiac index (CI—cardiac output/body surface), in L min^−1^ m^−2^, was obtained through the formula: stroke volume (SV) X heart rate where the SV is the result of the VTI (velocity time integral of the aortic flow obtained at the transgastric view) X CSA (aorta cross-sectional area obtained by the planimetry of the aortic valve at the transversal caudal view) (23). These variables were obtained by esophageal echocardiography (6Tc-RS TEE KN100104, Vivid Q; General Electric Health Care, IL, USA) and the body surface of each dog according to Haskins et al. (24).

In case of hypotension (MAP < 65 mmHg), the treatment was instituted with a lactated Ringer's solution fluid challenge (15 mL kg^−1^ for 15 min). If hypotension persisted, ephedrine was administered (Efedrin; Cristália LTDA, Brazil), in bolus 0.1–0.25 mg kg^−1^. If the MAP did not return to normal values even after ephedrine, the ventilation protocol was interrupted, and the patient was excluded from the study. The consumption of fluid and ephedrine were calculated in mL kg^−1^ and mg kg^−1^, respectively, at the end of the anesthetic procedure.

Mechanical ventilation was started using the volume-controlled ventilation mode (VCV) with a V_T_ of 8 mL kg^−1^, inspiratory: expiratory ratio of 1:2, and *f*_R_ adjusted to maintain PE'CO_2_ between 35 and 45 mmHg (4.7–6.0 kPa). The PEEP values were determined according to each group.

The V_T_, *P*_plat_, peak inspiratory pressure (PIP), and PEEP were measured using a sensor for inspiratory, and expiratory flow and volume measurements located between the endotracheal tube and the Y piece and connected to dedicated software for data display in a multiparametric data collection system (DX 2020; Dixtal, Brazil). The static compliance of the respiratory system (C_st_), alveolar oxygen tension (PAO_2_), alveolar-arterial oxygen tension difference [P(A-a)O_2_], the physiologic right-to-left shunt fraction (*Q*_s_/*Q*_t_), the alveolar dead space-tidal volume ratio (V_D_/V_T_) and driving pressure (DP) were calculated according to the following standard formulas:

C_st_ (mL cmH_2_O^−1^ kg^−1^) = [V_exp_/(P_PLAT_ – PEEP)]/body weight; (21)
V_D_/V_T_ (%) = (PaCO_2_ – EtCO_2_)/PaCO_2;_ (25)
*Q*_s_/*Q*_t_ (%) = [P(A-a)O_2_ × 0.003]/{4+[P(A-a)O_2_ × 0.003]}
*0.003 = oxygen solubility factor in total blood; (26)
Oxygenation index was acquired through formula PaO_2_/FIO_2_.
PAO_2_ (mmHg) = [FiO_2_ × (Pb – PH_2_O)] – [PaCO_2_/R)],
Pb = ambient barometric pressure (760 mmHg); PH_2_O is the partial pressure of water inside the respiratory system (47 mmHg), and R is respiratory quotient (0.8); (27)
P(A-a)O_2_ (mmHg) = PAO_2_ – PaO_2_
DP = *P*_plat_ – PEEP (28).

After weaning from mechanical ventilation, the neuromuscular blocker was reversed by neostigmine 0.04 mg kg^−1^ (Normastig; BiolabSanus LTDA, Brazil) associated with atropine 0.04 mg kg^−1^ (Pasmodex; Isofarma LTDA, Brazil) IV when TOF > 90% was observed. Extubation was performed when constant and repetitive swallowing reflexes were observed. Animals were allowed to recover from anesthesia in the recovery room. They were continuously monitored for HR, MAP, *f*_R_, rectal temperature and assessed by blood gas analysis and visual pain scale. They were discharged when alert, normothermic, and without pain.

### Study Design

Randomization was carried out before the study using the website^2^ to ensure the blind distribution of the dogs between groups. Group information was placed within individual manila envelopes that were open consecutively before the anesthesia procedure.

The VCV mode was started immediately after induction of anesthesia in all dogs. The dogs were randomly assigned into two groups, 11 dogs in the ARM group and 10 in the PEEP group. In the ARM group, the technique used to apply the maneuver was PEEP titration. The ventilation started with zero end-expiratory pressure (ZEEP), and PEEP was increased to 5, 10, and 15 cmH_2_O, followed by its decrease to 10 and 5 cmH_2_O, with a duration of 3 min in each level and a total ARM time of 15 min (ARM15). The 5 cmH_2_O PEEP was maintained until the end of the anesthetic procedure. In the PEEP group, the PEEP of 5 cmH_2_O was instituted from the beginning of anesthesia and kept throughout the study.

Cardiopulmonary data and arterial blood samples were obtained after 15 min of the beginning of anesthesia (baseline moment—BL) and 15 (ARM15/PEEP15), 45 (ARM45/PEEP45) and 75 min (ARM75/PEEP75) after BL (ARM-BL/PEEP-BL). The ARM 15 was collected immediately after finishing the maneuver. Moreover, arterial blood was collected during recovery of anesthesia at 5 (R5), 15 (R15), and 25 (R25) min after patient extubation.

### Statistical Analysis

All continuous numerical data are presented as mean ± standard deviation (SD). The normality assumption was verified using the Shapiro-Wilk test, and two-way analysis of variance with repeated measures was used to analyze treatment effects and interaction. *Post-hoc* analysis was performed with the Tukey test to identify differences among times points and Bonferroni test between treatments. Statistical significance was attributed when *p* < 0.05. The analyses were carried out using GraphPad Prism 6 (GraphPad Software, Inc., CA, USA).

## Results

A total of 22 dogs were included. However, one dog was excluded from the study due to hemodynamic instability that did not respond to the proposed treatment. There were no significant differences among groups in age (ARM group, 5.36 ± 1.86 years; PEEP group, 5.1 ± 1.9 years), body weight (ARM group, 14.55 ± 3.09 kg; PEEP group, 15.48 ± 3.28 kg), and total anesthesia time (ARM group, 107 ± 21.02 min; PEEP group, 114 ± 18.97 min).

### Hemodynamic Variables

In arterial pressure values, SAP, MAP and DAP were significantly higher in PEEP45 (*p* = 0.049; *p* = 0.007; *p* = 0.012, respectively) and PEEP75 (*p* = 0.018; *p* = 0.002; *p* = 0.016, in order) than in PEEP-BL (Table 1). The CI was significantly lower in the PEEP15 compared to ARM15 (*p* = 0.02). No significant changes were observed in other variables within a group or between groups (Table 1).

**Table 1:** Measurements of cardiovascular variables, ventilation and respiratory mechanics in isoflurane-anesthetized dogs mechanically ventilated with tidal volume of 8 mL kg^−1^ submitted to recruitment maneuver (ARM group, *n* = 11) or without recruitment maneuver (PEEP group, *n* = 10) and use of 5 cm H_2_0 of positive end expiratory pressure (PEEP).

**Variable**	**Group**	**PEEP-BL**	**PEEP15**	**PEEP45**	**PEEP75**
		**ARM-BL**	**ARM15**	**ARM45**	**ARM75**
HR	PEEP	114.7 ± 17.9	105.4 ± 15.9	114.5 ± 15.2	115.1 ± 22.1
(bpm)	ARM	108.7 ± 21.2	118.5 ± 27	126.8 ± 15.5	120.7 ± 19.3
SAP	PEEP	89.2 ± 17.6	99 ± 13.2	103.8 ± 15.9^*^	105.9 ±13.6^*^
(mmHg)	ARM	101 ± 27.5	102.7 ± 12.4	103.6 ± 16.5	101.5 ± 12.7
MAP	PEEP	58.8 ± 9.49	65.5 ± 10.1	71.5 ± 10.46^*^	73.1 ± 12.6^*^
(mmHg)	ARM	67.4 ± 12.4	72.4 ± 7.9	68.6 ± 7.8	67.5 ± 9.7
DAP	PEEP	47.5 ± 8.7	54.2 ± 9.9	59.4 ± 9.45^*^	59 ± 11.2^*^
(mmHg)	ARM	56.3 ± 11.3	60.9 ± 8.8	54.7 ± 7.3	54.3 ± 10.3
CI	PEEP	2.4 ± 0.6	2.2 ± 0.5^†^	2.6 ± 0.7	2.8 ± 0.4
(L min^−1^m^−2^)	ARM	2.6 ± 0.4	2.8 ± 0.4	3.0 ± 0.6	3.1 ± 0.5
*f*R	PEEP	14.3 ± 1.8	14.1 ± 2.0	15.6 ± 3.8	15.3 ± 3.7
(mpm)	ARM	15.3 ± 1.5	15.4 ± 2.3	16.4 ± 2.3	16.4 ± 3.1
PIP	PEEP	11.7 ± 0.8	11.9 ± 1	12.0 ± 1.1	11.8 ± 2.4
(cmH_2_0)	ARM	7.9 ± 1.8^†^	10.6 ± 0.9^*^	11 ± 0.8^*^	11.4 ± 1.1^*^
*P*_plat_	PEEP	10.7 ± 0.8	10.8 ± 1	11 ± 1.1	11.4 ± 1.2
(cmH_2_0)	ARM	6.9 ± 1.8^†^	9.6 ± 0.9^*^	10 ± 0.8^*^	10.4 ± 1.1^*^
DP	PEEP	5.7 ± 0.8	5.8 ± 1.0	6.0 ± 1.1	6.4 ± 1.3^*^
(cmH_2_0)	ARM	6.9 ± 1.8	4.6 ± 0.9^*^	5.0 ± 0.8^*^	5.4 ± 1.1^*^
C_st_	PEEP	1.46 ± 0.23	1.37 ± 0.25	1.39 ± 0.25	1.28 ± 0.58
(mL cmH_2_0^−1^ kg^−1^)	ARM	1.45 ± 0.37	1.72 ± 0.27^*^	1.63 ± 0.26	1.55 ± 0.29
Dead space	PEEP	9.4 ± 5.7^§^	11.4 ± 7.3^§^	12.1 ± 4.5^§^	18.5 ± 4.7
V_D_/V_T_ (%)	ARM	10 ± 2.9	12.5 ± 4.6	16.1 ± 4.9^*^	15.1 ± 8.2
Shunt fraction	PEEP	5.3 ± 2.4	5.1 ± 2.3	3.8 ± 2.5	3.9 ± 3.3
*Q*_s_/*Q*_t_ (%)	ARM	5.5 ± 1.6	3.4 ± 2.4^*^	2.9 ± 1.2^*^	4.3 ± 1.6
PE'CO_2_ (mmHg) (kPa)	PEEP	35.4 ± 2.4	35 ± 3.3^†^	35.2 ± 2.4	33.8 ± 2.9
		(4.7 ± 0.3)	(4.7 ± 0.4^†^)	(4.7 ± 0.3)	(4.5 ± 0.4)
	ARM	37.7 ± 3	41.5 ± 4.4^*^	38.5 ± 7	36.1 ± 4.5
		(5.0 ± 0.4)	(5.5 ± 0.6*)	(5.1 ± 0.9)	(4.8 ± 0.6)
PaCO_2_	PEEP	38.1 ± 2.3	38.8 ± 4.7^†^	39.1 ± 5.8	40.2 ± 5.5
(mmHg)	ARM	37.2 ± 12.6	46.0 ± 4.8^*^	42.5 ± 3.8^*^	42.6 ± 4.6^*^
SaO_2_	PEEP	99.8 ± 0.06	99.7 ± 0.26	99.8 ± 0.05	99.8 ± 0.07
(%)	ARM	99.7 ± 0.09	99.7 ± 0.09	99.8 ± 0.08	99.7 ± 0.09
PaO_2_/FIO_2_	PEEP	500.4 ± 42.3	496.7 ± 39.7	532.2 ± 45.8	529.7 ± 65.2
(mmHg)	ARM	461.2 ± 41	505.6 ± 59.2^*^	537.2 ± 33^*^	492.7 ± 46
PAO_2_	PEEP	368.0 ± 63.8	354.0 ± 68.4	354.8 ± 53	358.2 ± 62.6
(mmHg)	ARM	317.7 ± 20.9	301.0 ± 24	324.1 ± 13.9	316.1 ± 24
P(A-a)O_2_	PEEP	79.5 ± 35	74.4 ± 36.7	56.7 ± 37	58.1 ± 51.3
(mmHg)	ARM	78.9 ± 24	47.7 ± 33.7^*^	39.8 ± 17.6^*^	61.0 ± 23.5
${\text{HCO}}_{3}^{-}$	PEEP	20 ± 1.3	19.6 ± 1.8	19.4 ± 2.6	19.9 ± 2.4
(mmol/L)	ARM	18.3 ± 6.3	20.0 ± 1.6	19.9 ± 1.5	20.2 ± 1.2
BE	PEEP	−5.3 ± 1.4	−5.9 ± 1.5	−6.1 ± 2.3	−5.7 ± 2.2
	ARM	−5.2 ± 2.3	−6.9 ± 1.5	−6.4 ± 1.2	−5.9 ± 1.4
pH	PEEP	7.33 ± 0.02	7.32 ± 0.02^†^	7.32 ± 0.02	7.31 ± 0.02
	ARM	7.31 ± 0.02^‡^	7.25 ± 0.02	7.28 ± 0.04^‡^	7.29 ± 0.04^‡^
Rectal temp	PEEP	37.5 ± 0.5	37.4 ± 0.6	36.5 ± 0.8	35.7 ± 0.9
(°C)	ARM	37.7 ± 0.7	37.4 ± 0.9	36.6 ± 1.0	36.0 ± 1.2

*Data are mean ± standard deviation*.

*HR, heart rate; SAP, systolic arterial pressure; MAP, mean arterial pressure; DAP, diastolic arterial pressure; CI, cardiac index; f_R_, respiratory frequency; PIP, peak inspiratory pressure; P_plat_, plateau pressure; DP, driving pressure; C_st_, static compliance; V_D_/V_T_, dead space; Q_s_/Q_t_, shunt fraction; PE'CO_2_, end-tidal carbon dioxide; PaCO_2_, arterial partial pressure of carbon dioxide; SaO_2_, arterial oxygen saturation; PaO_2_, arterial partial pressure of oxygen; PaO_2_/FIO_2_, oxygenation index; PAO_2_, alveolar oxygen tension; P(A-a)O_2_, alveolar-arterial oxygen difference; ${\text{HCO}}_{3}^{-}$, blood bicarbonate; BE, base excess; Rectal temp, rectal temperature*.

† *Significantly different from ARM group (p < 0.05)*.

* *Significantly difference from BL within a group (p < 0.05)*.

§ *Significantly different from PEEP75 (p < 0.05)*.

‡ *Significantly different from ARM15 (p < 0.05)*.

Total fluid administered was 8.4 ± 3.7 mL kg^−1^ h^−1^ in the PEEP group and 12.0 ± 5.7 mL kg^−1^ h^−1^ in the ARM group. The ephedrine consumption was 0.07 ± 0.16 mg kg^−1^ in the PEEP group and 0.07 ± 0.11 mg kg^−1^ in the ARM group, with no differences between the two groups.

### Mechanical Ventilation

PIP and *P*_plat_ were higher in ARM15, ARM45, and ARM75 than ARM-BL (*p* < 0.0001). PIP and *P*_plat_ were significantly different between PEEP-BL and ARM-BL (*p* < 0.0001).

The DP in ARM-BL was higher than ARM15, ARM45, and ARM75 (*p* < 0.0002); for the PEEP group the DP was significantly different between PEEP-BL and PEEP75 (*p* = 0.036).

C_st_ improved in ARM15 compared with ARM-BL (*p* = 0.037). The V_D_/V_T_ in ARM45 was significantly higher than ARM-BL (*p* = 0.024). PEEP75 was higher than PEEP-BL, PEEP15, and PEEP45 (*p* < 0.05).

Shunt fractions in ARM15 and ARM45 were significantly smaller than ARM-BL (*p* < 0.01; Table 1).

### Gas Exchange

The PE'CO_2_ and PaCO_2_ were significantly higher in the ARM group than the PEEP group at 15 min (*p* = 0.002 and *p* = 0.04, respectively).

In relation to the difference between moments, at ARM15 the PE'CO_2_ was higher than ARM-BL (*p* = 0.0274) and ARM75 (*p* = 0.0007). PaCO_2_ in ARM-BL was lower than ARM15 (*p* = 0.0002), ARM45 (*p* = 0.047) and ARM75 (*p* = 0.038; Table 1). There was no difference between time points in the PEEP group.

PaO_2_/FIO_2_ ratio was significantly increased in ARM15 (*p* = 0.045) and ARM45 compared with ARM-BL (*p* < 0.0001). The P(A-a)O_2_ was smaller in ARM15 (*p* = 0.006) and ARM45 (*p* = 0.0004) than ARM-BL (Table 1). In the PEEP group and between groups, there was no difference.

### Metabolic Parameters

The pH was significantly higher in PEEP15 than ARM15 (*p* < 0.05). The pH in ARM15 was lower when compared to ARM-BL, ARM45, and ARM75 (*p* < 0.05; Table 1).

### Recovery From Anesthesia

Recovery was uneventful and desaturation was not observed. The PaO_2_ in ARM-R5 was higher than ARM-R15 (*p* = 0.0069) and ARM-R25 (*p* = 0.005). In the PEEP group, PEEP-R5 was higher also than PEEP-R25 (*p* = 0.0046; Table 2).

**Table 2:** Blood gas measurements during 5–25 min of recovery from isoflurane-anesthetized dogs mechanically ventilated with tidal volume of 8 mL kg^−1^ submitted to recruitment maneuver (ARM group, *n* = *11*) or without recruitment maneuver (PEEP group, *n* = *10*) and use of 5 cmH_2_O of positive end-expiratory pressure (PEEP).

**Variables**	**Groups**	**PEEP-R5**	**PEEP-R15**	**PEEP-R25**
		**ARM-R5**	**ARM-R15**	**ARM-R25**
pH	PEEP	7.37 ± 0.03	7.36 ± 0.02	7.35 ± 0.03
	ARM	7.35 ± 0.02	7.35 ± 0.03	7.36 ± 0.03
PaCO_2_	PEEP	34.8 ± 3.8	36.2 ± 4.4	38.0 ± 4.1^*^
(mmHg)	ARM	37.3 ± 3.3	38.3 ± 4.1	36.8 ± 4.4
PaO_2_	PEEP	122.6 ± 15.3	111.9 ± 12.3	106.6 ± 9.0^*^
(mmHg)	ARM	117.1 ± 13.4	106.2 ± 9.2^*^	107.7 ± 8.8^*^
SaO_2_	PEEP	98.3 ± 0.8	97.8 ± 0.9	97.6 ± 0.7
(%)	ARM	98.1 ± 0.4	97.5 ± 0.8	97.7 ± 0.8
P(A-a)O_2_	PEEP	−16.4 ± 14.9	−7.5 ± 8.8	−4.4 ± 7.8
(mmHg)	ARM	−14.0 ± 14.2	−4.4 ± 11.2	−3.9 ± 11.6

*Data are mean ± standard deviation.*

*PaCO_2_, arterial partial pressure of carbon dioxide; PaO_2_, arterial partial pressure of oxygen; SaO_2_, arterial oxygen saturation; P(A-a)O_2_, alveolar-arterial oxygen difference;*

* *significantly difference from R5 within a group (p < 0.05); PAO_2_ (mmHg) = [FiO_2_ x (Pb -PH_2_O)]-(PaCO_2_/R), where Pb = ambient barometric pressure (760 mmHg), PH_2_O = partial pressure of water inside the respiratory system (47 mmHg) and R = respiratory quotient (0, 8)*.

The telephone contact with the owners of the dogs involved in the study for 7 days after anesthesia; none reported seeing clinical changes or any sign of respiratory discomfort in the dogs.

## Discussion

The tidal volume of 8 mL kg^−1^ in healthy dogs undergoing mechanical ventilation with the application of PEEP associated with 50% FIO_2_ was shown to be effective for the maintenance of gas exchange and oxygenation without affecting hemodynamic variables throughout the procedure.

The suggested V_T_ for dogs ranges from 10 to 15 mL kg^−1^, except for animals with pulmonary injury, where 8 mL kg^−1^ is the value applied (16, 17). The use of low V_T_ in dogs continues to be controversial and has been disregarded because it is often associated with hypercapnia, acidosis, and hypoxemia. A study ruled out the benefits of applying the 8 mL kg^−1^ V_T_ associated with PEEP 5 cmH_2_O due to the occurrence of hypercapnia (54–60 mmHg, 7.2–8 kPa) and the need for a high *f*_R_ (above 30 mpm) (19). In the study of Bumbacher et al. (17), in which V_T_ of 10, 12, and 15 mL kg^−1^ were compared, the authors concluded that this last one was the most efficient to ventilate the animals and was not associated with pulmonary lesions in the patients at the time of the study (17). However, complications in the post-anesthetic period were not evaluated, and animals in dorsal recumbency were not considered. Furthermore, according to the authors, pro-inflammatory mediators that could have shown alveolar structure changes and inflammation due to high V_T_ were not measured.

Considering the occurrence of hypercapnia and tachypnea in the present study, the PEEP group did not show these alterations. The highest PE'CO_2_ (41.5 ± 4.4 mmHg, 5.5 ± 0.6 kPa) and PaCO_2_ (46.0 ± 4.8 mmHg) and lowest pH values (7.25 ± 0.02) were observed at the end of ARM and were lower than those observed in the cited studies. It can be explained by a decrease in the alveolar concentration of CO_2_ due to the increase in functional residual capacity and less elimination. However, the use of PEEP tends to reduce the expiratory volume and increase dead space. Besides that, an increased transthoracic pressure decreases venous return and holds CO_2_ produced by tissues in peripheral blood vessels, raising its concentration in the blood (21, 29, 30). Oura et al. (31) evaluated the use of V_T_ 6 to 15 mL kg^−1^ and they observed V_T_ 6 mL kg^−1^ was enough to ventilate dogs, in addition to hypercapnia (PE'CO_2_ 40.4 ± 2.2 mmHg, 5.39 ± 0.29 kPa) and mild respiratory acidosis (pH 7.25 ± 0.02).

Permissive hypercapnia is accepted in human patients kept in protective mechanical ventilation, the reason why in our study, the higher values of PE'CO_2_ were allowed. This standard had the advantage of protecting pulmonary function and minimizing the depressive effects of mechanical ventilation on cardiovascular function due to its sympathomimetic effect (27). Another point to be considered is that the degree of hypercapnia observed in this study can be easily minimized by increasing the respiratory rate. We chose to maintain the average values of around 15 movements per min to verify the magnitude of the changes in the PaCO_2_. Still, it can be increased without prejudice to the patient and within the physiological range.

The hypercapnia and respiratory acidosis in the ARM group quickly reversed with the return of spontaneous ventilation. Adequate gas exchange and oxygenation were observed during anesthetic recovery, even without oxygen supplementation. After the patients were released, telephone contact was maintained for 7 days after the study was carried out, and no post-anesthetic complications related to the respiratory system were reported which was also in concurrence with findings of by Karalapillai et al. (32). These authors evaluated the use of protective ventilation in humans with V_T_ of 6 mL kg^−1^ and conventional ventilation with V_T_ of 10 mL kg^−1^. All patients were anesthetized for 2 h and underwent different surgical procedures (except intrathoracic and intracranial). Within a period of up to 7 days after surgery, no statistical differences were observed between these groups on the occurrence of primary pulmonary complications such as pneumonia, bronchospasm, and atelectasis, and secondary, such as embolism, acute respiratory distress syndrome, and sepsis.

Concerning the use of PEEP associated with the ARM, it was not superior to the isolated use of PEEP in this study. ARM improved oxygenation and C_st_ and reduced the shunt fraction, P(A-a)O_2_, and DP as expected (18, 20, 33–37). However, the application of PEEP since the anesthetic induction promoted similar values to the ARM group, which were sustained during all the procedures. The V_D_/V_T_ values were similar in both groups and remained within the normal range for the species (25, 38). Therefore, there was no suspicion that PEEP promoted hyperventilation of the non-perfused areas. The success in the single use of PEEP to maintain ventilation and oxygenation depends on the amount of pressure used and the levels needed to open and close the lungs in each patient (39). These values are individual, and there is still no consensus on ideal values. In the present study, all animals had the same body composition score (4–6) and relatively low weight (10–20 kg), which probably contributed to the results observed. For heavier animals or those kept in dorsal recumbency, a PEEP of 5 cmH_2_0 may not be enough. In these situations, the PEEP value should be titrated. For this reason, the use of low V_T_ and an adequate PEEP level can maintain adequate ventilation and oxygenation. The strategy of pulmonary protective ventilation is known.

The established mechanical ventilation protocols, with and without ARM, resulted in low DP values. These lowest DP values were related to a better prognosis in ventilated patients with lung injury, which also indicates a protective strategy applied to the patients in our study (28, 40, 41).

We identified that there was no impact on hemodynamic variables after ARM. Consequently, there was no difference between the groups concerning the fluid administered and the need for vasoactive drugs. Hypotension observed at the beginning of the procedure probably was secondary to anesthetic induction, so it was treated, and there was no recurrence 15 min later. Although ARM can compromise patients' hemodynamics, promoting transitional hypotension and tachycardia (42), we have not identified it in our patients.

A limiting factor in this study was the evaluation time. More prolonged procedures could change the results because using PEEP alone would not be enough to avoid new atelectasis formation. Another limitation is the fact that patients with healthy lungs were studied. Protective ventilation is adequate for injured lungs, making it challenging to have discrepant results between the two strategies, as they were healthy lungs with high adaptive capacity. In future studies, the acquisition of lung images by computed tomography, electrical impedance tomography, and ultrasound can provide more accurate information on the distribution of ventilation performed with these low volumes and the number of areas that remain closed and open with the performance of alveolar recruitment maneuvers or only PEEP use.

In conclusion, using 8 mL kg^−1^ V_T_ and PEEP of 5 cmH_2_O effectively promoted adequate gas exchange. Despite using ARM and PEEP improved oxygenation and C_st_ and reduced the shunt fraction and driving pressure without impact on the cardiovascular system, the group of isolated PEEP showed good values in all variables. This study indicates that protective mechanical ventilation was adequate for 1 h of dental surgery with or without recruitment maneuvers.

## Data Availability Statement

The raw data supporting the conclusions of this article will be made available by the authors, without undue reservation.

## Ethics Statement

The animal study was reviewed and approved by Animal Use Ethics Committee of College of Veterinary Medicine and Animal Science of University of São Paulo, approved the experimental protocol under Protocol No. 3581110716. Written informed consent was obtained from the owners for the participation of their animals in this study.

## Author Contributions

RR: study design, data acquisition, analysis and interpretation of data, and drafted the manuscript. LG: study design, data acquisition, and analysis and interpretation of data. PV: study design and data acquisition. AE and AS: drafted the manuscript. AA and DF: conceived the study, study design, advice on data analysis and interpretation, and manuscript revision. All authors contributed substantially to manuscript revision and approved the final manuscript.

## Funding

This study was financed in part by the Coordenação de Aperfeiçoamento de Pessoal de Nível Superior—Brasil (CAPES)—Finance Code 001.

## Conflict of Interest

The authors declare that the research was conducted in the absence of any commercial or financial relationships that could be construed as a potential conflict of interest.

## Publisher's Note

All claims expressed in this article are solely those of the authors and do not necessarily represent those of their affiliated organizations, or those of the publisher, the editors and the reviewers. Any product that may be evaluated in this article, or claim that may be made by its manufacturer, is not guaranteed or endorsed by the publisher.

## Footnotes

^1^ https://www.sealedenvelope.com

^2^ www.randomization.com
