# Corrigendum: Intraoperative protective mechanical ventilation in dogs: A randomized clinical trial

**DOI:** 10.3389/fvets.2022.1050451

**Published:** 2022-11-28

**Authors:** Renata R. Rodrigues, Aline M. Ambrósio, Aline M. Engbruch, Lucas A. Gonçalves, Paula A. Villela, Ana F. Sanchez, Denise T. Fantoni

**Affiliations:** Department of Surgery, Faculty of Veterinary Medicine and Animal Science, University of São Paulo, São Paulo, Brazil

**Keywords:** mechanical ventilation, low tidal volume, alveolar recruitment maneuver, positive end-expiratory pressure, gas exchange, ventilatory mechanics, hemodynamics, dogs

In the published article, there was a typing error in the data of P_(A−a)_O_2_ in Table 2. The corrected Table 2 and its caption appear below.

**Table 2:** Blood gas measurements during 5–25 min of recovery from isoflurane-anesthetized dogs mechanically ventilated with tidal volume of 8 mL kg^−1^ submitted to recruitment maneuver (ARM group, *n* = *11*) or without recruitment maneuver (PEEP group, *n* = *10*) and use of 5 cmH_2_O of positive end-expiratory pressure (PEEP).

**Variables**	**Groups**	**PEEP-R5**	**PEEP-R15**	**PEEP-R25**
		**ARM-R5**	**ARM-R15**	**ARM-R25**
pH	PEEP	7.37 ± 0.03	7.36 ± 0.02	7.35 ± 0.03
	ARM	7.35 ± 0.02	7.35 ± 0.03	7.36 ± 0.03
PaCO_2_	PEEP	34.8 ± 3.8	36.2 ± 4.4	38.0 ± 4.1^*^
(mmHg)	ARM	37.3 ± 3.3	38.3 ± 4.1	36.8 ± 4.4
PaO_2_	PEEP	122.6 ± 15.3	111.9 ± 12.3	106.6 ± 9.0^*^
(mmHg)	ARM	117.1 ± 13.4	106.2 ± 9.2^*^	107.7 ± 8.8^*^
SaO_2_	PEEP	98.3 ± 0.8	97.8 ± 0.9	97.6 ± 0.7
(%)	ARM	98.1 ± 0.4	97.5 ± 0.8	97.7 ± 0.8
P(A-a)O_2_	PEEP	−16.4 ± 14.9	−7.5 ± 8.8	−4.4 ± 7.8
(mmHg)	ARM	−14.0 ± 14.2	−4.4 ± 11.2	−3.9 ± 11.6

Data are mean ± standard deviation.

PaCO_2_, arterial partial pressure of carbon dioxide; PaO_2_, arterial partial pressure of oxygen; SaO_2_, arterial oxygen saturation; P(A-a)O_2_, alveolar-arterial oxygen difference;

* , significantly difference from R5 within a group (p < 0.05); PAO_2_ (mmHg) = [FiO_2_ x (Pb -PH_2_O)]-(PaCO_2_/R), where Pb = ambient barometric pressure (760 mmHg), PH_2_O = partial pressure of water inside the respiratory system (47 mmHg) and R = respiratory quotient (0, 8).

The authors apologize for this error and state that this does not change the scientific conclusions of the article in any way. The original article has been updated.

## Publisher's note

All claims expressed in this article are solely those of the authors and do not necessarily represent those of their affiliated organizations, or those of the publisher, the editors and the reviewers. Any product that may be evaluated in this article, or claim that may be made by its manufacturer, is not guaranteed or endorsed by the publisher.

